# Early socioeconomic conditions to children’s trait resilience: longitudinal mediation effects of mothers’ and fathers’ parenting

**DOI:** 10.1186/s13034-025-00979-1

**Published:** 2025-11-10

**Authors:** Meryl Yu, Germaine Y. Q. Tng, Zhan Lin, Helen Chen, Johan Gunnar Eriksson, Yap Seng Chong, Henning Tiemeier, Peipei Setoh

**Affiliations:** 1 Department of Psychology, National University of Singapore, Singapore, Singapore; 2 Division of Psychology, School of Social Sciences, Nanyang Technological University, 48 Nanyang Avenue, Singapore, 639818 Singapore; 3 Department of Psychological Medicine, KK Women’s and Children’s Hospital, Singapore, Singapore; 4 Duke-NUS Medical School, Singapore, Singapore; 5 A*STAR Institute for Human Development and Potential, Singapore, Singapore; 6 Department of Obstetrics and Gynaecology, Yong Loo Lin School of Medicine, National University of Singapore, Singapore, Singapore; 7 Department of Social and Behavioral Sciences, Harvard T.H. Chan School of Public Health, Boston, Massachusetts, USA; 8 Yong Loo Lin School of Medicine, National University of Singapore, Singapore, Singapore

**Keywords:** Resilience, Mothers, Fathers, Parenting, Childhood, Socioeconomic status, Culture

## Abstract

**Background:**

Trait resilience is a well-established protective factor against diverse social-emotional challenges and stress-associated psychiatric disorders. Its cultivation may thus be exceptionally critical for children exposed to early financial adversity, which has been demonstrated to confer elevated vulnerabilities to psychosocial maladjustment. Grounded in the Family Stress Model with a focus on an understudied Asian population, this longitudinal study investigates how early family socioeconomic disadvantage indirectly shapes children’s trait resilience through key parenting dimensions—warmth, rejection, and autonomy support—by both mothers and fathers.

**Methods:**

This longitudinal study was embedded in a multi-ethnic, pre-birth cohort involving Singaporean families (57.3% Chinese, 30.0% Malay, 12.7% Indian), comprising 430 biological mothers (*M*_Age_ = 30.5, *SD*_Age_ = 5.13), 430 children (47.9% female), and 348 biological fathers (*M*_Age_ = 33.9, *SD*_Age_ = 6.03). Key family socioeconomic characteristics were measured at baseline during the 11th week of pregnancy via maternal report (maternal education, household income, and housing type), and at 2 or 3 years postnatal via paternal report (paternal education). Children reported on mothers’ and fathers’ parenting practices (warmth, rejection, and autonomy support) separately at 8.5 years with the *Parental Bonding Instrument*, and trait resilience at 10.5 years with the *Connor-Davidson Resilience Scale-25*.

**Results:**

Children from families with lower socioeconomic status during early childhood (indexed by lower levels of mothers’ and fathers’ educational attainment, and household income) demonstrated lower trait resilience in late childhood. Parallel mediation analyses with 5,000 bootstrapped samples revealed that maternal and paternal educational attainment influenced trait resilience in late childhood via greater maternal and paternal rejection during middle childhood, respectively. Meanwhile, the relationship of household income with children’s trait resilience was mediated by lower levels of maternal warmth only. No significant indirect effects of both parents’ autonomy support were observed.

**Conclusions:**

Maternal and paternal parenting practices play salient roles in nurturing children’s trait resilience, in part substantiating the cultural validity of the Family Stress Model within a Southeast Asian family ecology. Specifically, family interventions could seek to ameliorate both maternal and paternal rejection, as well as enhance maternal warmth behaviors to mitigate the influence of socioeconomic disadvantage on children’s trait resilience.

**Supplementary Information:**

The online version contains supplementary material available at 10.1186/s13034-025-00979-1.

## Background

Early socioeconomic disadvantage confers cumulative risks over time, impacting children’s mental, emotional, and behavioral health trajectories [1]. Specifically, early socioeconomic hardship may attenuate children’s trait orientations to resilience by constraining access to supportive resources that can scaffold adaptive stress-response capacities [2]. Broadly, resilience refers to the successful withstanding and recovery from adversity [3] and has been recognized as a construct that integrates trait-level constancy and situational states [4]. *Trait resilience* is conceived to be a dispositional quality marked by enduring psychological strengths that confer differential susceptibility to bouncing back from sustained hardships [5]. By way of contrast, *resilience-as-a-state* reflects the momentary mobilization of resources to manage acute, situationally-specific disturbances (e.g., state resilience that is activated during work stress may not be transferable to manage family stress) [6, 7]. Another point of conceptual clarity lies in distinguishing the scope of resilience from tangential constructs, namely coping. Coping refers to an umbrella of specific cognitive-behavioral strategies to regulate one’s response to stressful scenarios [8]. While the idea of resilience presupposes successful and positive recovery, coping, conversely, need not be constructive, but can in fact be maladaptive in nature (e.g., other-blame, catastrophizing) [9]. Take rumination as one illustration. This coping response entails replaying and dwelling on the source of one’s distress; such repetitive thought cycles have been established to preclude active problem-solving, amplify negative emotions, and exacerbate vulnerability to disordered mental health [10]. Herein we adopt a trait-based perspective to investigate how enduring propensities in children’s trait resilience may prospectively emerge from early environmental influences.

Importantly, trait resilience is posited to offer transdiagnostic protection against stress-associated psychiatric disorders in children [11]. Further, children with higher trait resilience demonstrate better academic performance, social-emotional competencies, and self-regulation [12]. Altogether, empirical investigation into correlates of trait resilience thus has potential to inform the etiology, mitigation, and treatment of stress-induced psychopathology while enhancing positive adjustment in children.

Given an expansive protective value, an imperative research line is to understand the multi-factorial antecedents of trait resilience in children at-risk of maladaptation. While some research has examined neurocognitive correlates (e.g., stress activation, selective attention) of resilience in children from socioeconomically at-risk backgrounds [13], what is equally critical is to identify the intermediary environmental mechanisms in children’s proximal family environments. Although demonstrating modest rank-order stability over time [14, 15], empirical evidence suggest that trait resilience can in fact be amenable to enhancements [16]. Ascertaining family-level precursors is thus critical, as these could then serve as the focus for modification through targeted interventions from an early developmental stage.

### The relationship between early socioeconomic disadvantage and children’s resilience

Socioeconomic status (SES) is a multi-dimensional construct reflecting one’s level of access to social and financial resources [17]. In line with this conceptualization, SES has traditionally been measured as a composite of household income, level of education, housing assets, and occupational prestige [17]. Nevertheless, researchers of late have argued that socioeconomic conditions are not interchangeable and should not be aggregated into a unitary construct [18]. The latest recommended practice is to employ individual indicators (e.g., only income or education) based on the strength of empirical and theoretical relevance [19]. The present research adheres to these recommendations in our operationalization of SES, by first examining the zero-order associations of multiple SES markers with children’s resilience.

Importantly, meta-analytic studies have found that children raised under conditions of socioeconomic disadvantage tend to fare worse across a wide array of health and social-cognitive domains [1]. Despite vast interest in SES research, one understudied area is how early socioeconomic circumstances might socialize inter-individual differences in trait resilience [20]. Greater attention is thus warranted to disambiguate the ways socioeconomic antecedents set the context for children’s resilience development, and to understand the modifiable mechanisms that underpin this pathway.

### Parenting practices as a mediating mechanism

In elucidating the influence of early SES on children’s resilience, a pertinent theoretical framework is the Family Stress Model (FSM). The FSM posits that economic hardships place significant pressure on families by exacerbating parents’ child-rearing stress that, in turn, interfere with parents’ capacity for positive parenting practices that then bolster children’s vulnerabilities to maladaptation [21]. As an illustration, the chronic fatigue arising from struggling to place food on the table can leave scarcer emotional and cognitive energy available for supportive parenting [22]. Indeed, caregiving against the backdrop of financial hardship has been linked to diminished parental warmth [23], less parental autonomy support [24], and heightened parental rejection [25] that in turn have been implicated in children’s suboptimal social-emotional adjustment [25]. Recognized as one of the principal socializing agents in childhood, parents play an indispensable role during this formative period of social-emotional development marked by heightened neuroplasticity [26]. Disruptions to adaptive caregiving during this sensitive window can have long-lasting ramifications on children’s social-emotional skills: early caregiving critically shapes children’s internal working models of relationships [27], and these mental representations may consequently influence how children perceive, construe, and acclimate to everyday stressors in the social world [28]. Collectively, these empirical insights substantiate a sequential pathway whereby early socioeconomic disadvantage may lay the basis for the emergence of less adaptive parenting behaviors that, then, circumscribe children’s social-emotional development in concord with the FSM.

To strengthen the explanatory robustness of the FSM, there is increasing impetus to understand the model’s theoretical components within the frame of the *principles of commonality and specificity* [29]. These principles, built on cultural relativism, underscores the criticality of discerning parenting dimensions that are culturally invariant (i.e., common) from those whose expressions and developmental functions are culturally bounded (i.e., specific) [30, 31]. Meta-analyses spanning five continents demonstrated that children’s perceived parental warmth was panculturally predictive of better social-emotional development in terms of higher self-esteem and emotional stability [32, 33]. Researchers have taken such meta-analytic syntheses to argue that parental warmth may be a universally beneficial dimension of caregiving due to children’s fundamental desires to feel accepted by parents [34]. Notwithstanding this strong empirical foundation, the specific relationship of parental warmth with child trait resilience with Southeast Asian ecologies remains markedly underexamined.

The picture surrounding the universality of parental autonomy support is less clear. Foundational postulates of the Self-Determination Theory [35] position autonomy as a core psychological drive essential for healthy social-emotional development, with meta-analyses corroborating this proposition [36]. However, the relatively limited representation from Southeast Asian children hinders definitive conclusions, further suggesting a need to inspect cultural moderation in the developmental function of autonomy support. Of note, children within collectivistic societies are socialized to prioritize relational interdependence with parents (filial piety) [37]. Consequently, Asian children may not strive for autonomy to the same degree and in the same individuating manner as emphasized in Anglo-White frameworks [38]. This cultural moderation underscores the importance of empirical replication to ascertain whether parental autonomy support yields comparable developmental benefits for Southeast Asian children.

Meanwhile, there have been some assertions that parental rejection may be universally detrimental to child development [39], although some researchers have critiqued this perspective to be deterministic and overstated [25]. In a cross-cultural study of Arab, Indian, French, Polish, and Argentinian teenagers, Dwairy [25] reported that teenagers from Western (i.e., more individualistic) countries expressed less parental rejection compared to Eastern (i.e., more collectivistic) counterparts. Yet, even within Eastern cultures parental rejection was not uniformly predictive of adverse adjustment [25]. This within-cultural heterogeneity underscores a necessity to move beyond monolithic conceptions of the East, and to instead elucidate culturally granular patterns of parenting within specific Eastern societies [40]. Therefore, greater inclusion of Southeast Asian families would enrich the FSM’s capacity to reflect the diversity of caregiving process in the Majority World.

## The present study

Altogether, while prior investigations of the FSM have largely centered on children’s maladjustment, there remains a notable absence—and thus a key opportunity—in evaluating whether analogous family-level processes underlie positive development, namely trait resilience [41]. It is herein essential to recognize that resilience is not simply the inverse of dysfunction: resilience encompasses the presence of protective capacities rather than solely the absence of pathology or maladjustment [42]. This conceptual distinction underscores the cruciality of clarifying family processes that actively nurture resilience, rather than extrapolating conclusions from past research focused on maladaptive development. Another key contribution lies in extending and testing the tenets of the FSM to investigate both mother-child and father-child relationships within Southeast Asian families, a population that is comparatively understudied within the FSM literature [43]. 

To these ends, several research questions emerge. First, which specific socioeconomic indices are predictive of children’s trait resilience in Singapore’s ecology? Second, how do maternal and paternal parenting dimensions (warmth, rejection, and autonomy support) serve as intermediary mechanisms through which early socioeconomic conditions shape trait resilience in late childhood? Leveraging a pre-birth cohort design, we aimed to understand how early-life proxies of SES (maternal and paternal education, housing type, household income) would be tethered to children’s trait resilience at age 10.5 years. Building on this foundation, the role of maternal and paternal parenting measured during middle childhood (8.5 years old), along the specific dimensions of warmth, rejection, and autonomy support, were then evaluated as mediators in the pathway from early SES to children’s trait resilience.

In line with the FSM, we hypothesized that higher early-life SES would prospectively predict higher trait resilience at age 10.5 years, though we do not advance specific predictions about the relative salience of specific SES indices given the descriptive nature of this investigation. Meanwhile, we hypothesized that the pathway from higher SES to higher trait resilience would operate via the parenting mechanisms of greater parental warmth, greater autonomy support, and less rejection, congruent with past literature demonstrating the coupling of SES with these caregiving dimensions [25]. 

## Methods

### Study design and participants

The present longitudinal study was embedded within a larger pre-birth cohort study, Growing Up in Singapore Towards Healthy Outcomes (GUSTO). Expectant mothers aged ≥ 18 years, who were attending first trimester ultrasound scans at KK Women’s and Children’s Hospital and National University Hospital in Singapore were recruited from June 2009 to September 2010. Recruited mothers had to be Singaporean citizens or permanent residents, who planned to deliver at one of these hospitals, and had to continue residing in Singapore for the next five years. Both single and married mothers were eligible to take part. In keeping with Singapore’s ethnic composition, the fetuses had to be of Chinese, Indian, or Malay ethnicity, with ethnically homogenous parental ancestry (i.e., both set of grandparents had identical ethnicities). To augment statistical power for subgroup-specific analyses, ethnic minorities (i.e., Malays and Indians) were over-sampled. See Soh and colleagues’ study [44] for a detailed description.

Upon delivery, mother-child pairs were invited for continued waves of in-person neuropsychological assessments at key developmental stages: the neonatal period (weeks 1, 3, and 6), infancy (months 3, 6, 9, 15, 18), early childhood (annual visits from age 1 to 6 years, with another key timepoint at month 54), middle childhood (age 7, 8, 8.5, and 9 years), and continuing through early adolescence (annually from age 10 to 14 years, data collection ongoing). At every timepoint, translated questionnaires (in Mandarin Chinese, Malay, and Tamil) were available. These were prepared through a forward-backward translation process by language-proficient bilingual staff to safeguard cultural accuracy. Correspondingly, bilingual research coordinators (fluent in English and a second language i.e., Chinese, Malay, or Tamil) running the visits were trained to administer surveys based on parents’ language-of-preference. Visits were operated according to standardized protocols under oversight of GUSTO principal investigators to ensure procedural fidelity and were scheduled flexibly across both weekdays and weekends to accommodate work and school schedules. Data collection was conducted in private rooms within designated research units, housed at KK Hospital and the National University of Singapore, to minimize environmental distractions and uphold confidentiality. For all parent-reported measures, written informed consent was acquired. For all child-reported measures, written parental informed consent was taken, alongside the acquisition of written assent when children turned 7 years old.

For our specific research aims, we focused on children who had completed trait resilience measurement at the age 10.5 years wave. This focus yielded a subsequent analytic sample of 430 families (57.3% Chinese, 30.0% Malay, 12.7% Indian): 430 biological mothers (*M*_Age_ = 30.5, *SD*_Age_ = 5.13), 430 children (47.9% female), and a smaller subset of 348 biological fathers (*M*_Age_ = 33.9, *SD*_Age_ = 6.03). See Supplementary Table 1 for sample characteristics [Additional File 1].

Of note, GUSTO was originally conceived as a maternal-focused study to track mothers’ and children’s metabolic and neuropsychological health, with systematic early recruitment of pregnant women. The study protocol was expanded to recruit biological fathers only at a later phase, who were invited to take part when children were 2 years old. Consequently, paternal involvement represented a smaller, less consistent, and later-enrolling subset within the larger study population. To ascertain whether there was selection bias engendered by this enrollment process, we compared whether families *with* paternal participants (*N* = 348) structurally differed from counterparts *without* paternal participants (*N* = 82). Group comparison tests revealed no systematic differences of these families with respect to child gender, child ethnicity, maternal education, and all dimensions of paternal parenting (*p*s > 0.10). Nonetheless, families *without* available paternal participants were over-represented in the “<$2,000” income tier, and under-represented in the “S$4,000 to $5,999” (second highest) bracket (see Supplementary Analysis 1 and Supplementary Fig. 1 for full details [Additional File 2]). Thus, with the exception of household income, families *with* versus *without* paternal participants were matched on key socio-demographic and psychosocial traits.

## Measures

### Socioeconomic and demographic characteristics

Baseline maternal-reported socioeconomic and demographic characteristics were assessed during the in-person recruitment visit during 11th week of pregnancy. This comprised categorical information on biological mothers’ and biological fathers’ ethnicities (1 = *Chinese*, 2 = *Malay*, 3 = *Indian*) and the marital status (1 = *Married*, 0 = *Single*), as well as ordinal information on household monthly income (1 = < *S$2000*, 2 = *S$2000 to S$3999*, 3 = *S$4000 to S$599*9, 4 = >*S$6000*) and maternal educational attainment (1 = *Secondary or Below*, 2 = *Diploma or Certificates*, 3 = *College and Above*). Here, the category of “Secondary and Below” corresponded to the completion of secondary education or lower, roughly equivalent to a high school diploma in the American education system. Meanwhile, “Diploma and Certificates” represented post-secondary non-degree qualifications such as vocational diplomas, roughly comparable to community college or technical training diplomas. Meanwhile, the category “College and Above” denoted completion of a Bachelor’s, Master’s, or doctoral degree. Likewise, ordinal information on paternal educational attainment was reported by fathers using these identical categories at 2 or 3 years postnatal. Meanwhile, ordinal information on housing type was collected and organized as such: 1 = *1-*,* 2-*,*or 3-Room Public Flat*, 2 = *4- or 5-Room or Executive Public Flat*, 3 = *Private Property.* Housing type is a common proxy for SES when researching Singaporean samples [45]. In Singapore, 80% of the population reside in public flats, which are heavily subsidized [46]. Generally, smaller-sized public flats are priced more affordably compared to larger-sized ones. Meanwhile, private properties refer to residences such as bungalows and terrace houses that do not benefit from any state-sponsored financial grants. Accordingly, a larger ordinal number (e.g., 3 vs. 2, 2 vs. 1, 3 vs. 1) indexed higher property prices, delineating higher SES in Singapore’s housing market.

Birthdates of both biological parents were filled in by mothers during baseline recruitment, which were then calculated into continuous age scores. In anticipation of non-linear age effects (see scatterplots in Supplementary Figs. 2a and 2b [Additional File 3]) and to facilitate theoretical interpretability in understanding statistical associations in the relationship of parental age with children’s trait resilience, we re-coded the parental ages into three categories: 1 = *18 to 24 Years (Young Parents)*, 2 = *25 to 34 Years (Average Childrearing Ages)*, 3 = *35 Years and Above (Advanced Parental Age)*. These categorizations have been found to reflect salient developmental and socioeconomic distinctions by parental ages [47]. Namely, extant research demonstrates that young parents (18 to 24 years) tend to face more financial precarity, have more limited educational attainment, and more limited access to perinatal healthcare [48, 49]. Meanwhile, average childrearing ages (25 to 34 years) generally coincides with peak levels of health and labor force activity [50, 51]. Lastly, pregnancy at an advanced age (≥ 35 years) confers a duality of disadvantages and opportunities [52]: while older parents possess greater financial stability [53], they at the same time carry more adverse pregnancy risk [54]. These stratifications in parental ages thus constitute a theoretically pertinent framework for interrogating the ways in which parental sociodemographics may condition children’s trait resilience.

Meanwhile, categorical codes on child ethnicity (1 = *Chinese*, 2 = *Malay*, 3 = *Indian*) was derived from parental ethnicity. Nominal information on child sex (0 = *Female*, 1 = *Male*) was retrieved from birth records. Physical hardcopy checks verified that 96.74% of mothers completed the sociodemographic questionnaires in English. Meanwhile, administrative information denoted that 354 fathers (82.3%) preferred English, 54 preferred Mandarin Chinese (12.6%), 19 preferred Malay (4.4%), and 3 preferred Tamil (0.7%), reflecting the high English literacy rate in Singapore.

#### Parenting practices (Age 8.5 Years)

The original English version of the Parental Bonding Instrument (PBI) [55] was completed by children for mothers and fathers separately during the in-person visit at age 8.5 years. The PBI is one of the most widely utilized measures of parenting, with robust psychometric properties such as strong test-retest reliability [56] and construct validity in Southeast Asian samples [57]. Importantly, the PBI has demonstrated acceptable internal consistency in pediatric samples as young as 6 years old [58, 59]. Across 25 items, the PBI has four subscales, with six items reflecting parental warmth (e.g., “my father/mother is affectionate to me”), six items for parental rejection (e.g., “my father/mother makes me feel like I’m not wanted”), six items for parental autonomy support (e.g., “my father/mother lets me decide things on my own”), and seven items for parental overprotection (e.g., “my father/mother tries to control everything I do”). Items were rated on a 4-point Likert-type truth scale (0 = *Not True* to 3 = *Very True*). Item-level scores were summed into subscale scores, calculated for mothers and fathers separately. No item-level missing data was reported. With exception of the maternal and paternal overprotection subscales (Cronbach’s *α* = 0.65 for both), internal consistency of the warmth (Cronbach’s *α* = 0.80 and 0.83), rejection (Cronbach’s *α* = 0.75 and 0.70), and autonomy support subscales (Cronbach’s *α* = 0.75 and 0.76), for mothers and fathers respectively, were acceptable; the overprotection subscales were subsequently excluded from analyses.

#### Children’s trait resilience (Age 10.5 Years)

The Connor-Davidson Resilience Scale-25 (CD-RISC-25) is a widely validated, self-reported measure of trait resilience [5], commonly administered with children [60], counting those of Asian descent [61, 62]. As an illustration, the CD-RISC-25 had good internal consistency (Cronbach’s α = 0.86) in a Chinese sample of 6- to 16-year-old children (*M*_Age_= 11.7 Years, *SD*_Age_ = 2.18 Years) [61], as well as excellent internal consistency in the present sample (Cronbach’s *α* = 0.93).

Children completed the original English version of the CD-RISC-25 during the in-person study visit at age 10.5 years. The scale has 25 items, rated on a 4-point Likert scale from 1 (N*ot True at All*) to 4 (*True Nearly all the Time*). Items assessed perceived capacity to bounce back from adversity (e.g., “I can deal with whatever comes my way”). Of 430 children, only one respondent (0.23%) had one missing response out of 25 items (4%). Person mean imputation was employed to estimate the missing value, in line with Connor and Davidson’s recommendations [63]. Subsequently, item-level scores were summed, where a higher aggregate score reflected greater resilience.

### Data analytic plan

Analyses were conducted with R 4.4.1. We first ran Little’s test [64] for Missing Completely at Random (MCAR) with the full cohort sample to detect any non-random patterns of missingness. We then inspected the distribution of trait resilience to ascertain normality of scores for subsequent analyses.

Next, we examined relationships of children’s trait resilience with demographics (child sex and ethnicity, parents’ marital status, maternal and paternal age) to identify salient covariates. To ascertain key socioeconomic indicators tethered to children’s resilience [18], we then investigated zero-order relationships of SES characteristics with children’s resilience: maternal and paternal education, household income, and housing type. Given our central interest in SES, significant demographic factors were controlled for during this step to isolate the independent influence of SES. Here, independent samples *t*-tests were conducted for dichotomous factors (i.e., child sex and parents’ marital status); one-way analyses of variance (ANOVA) tests were conducted with categorical and ordinal variables with three or more levels (i.e., maternal and paternal education, household income, housing ownership, child ethnicity, maternal and paternal age). When *F*-tests detected significant group differences, post-hoc pairwise comparisons with Bonferroni’s Correction were ran to determine specific group contrasts driving the observed associations. For ordinal measures, polynomial contrast analysis was further run to assess the linearity of effects.

Finally, to identify plausible mechanisms in the sequence from early socioeconomic conditions (indexed by statistically significant SES characteristics) to children’s trait resilience, separate parallel mediation models were specified using *lavaan* [65] with maternal and paternal parenting practices as candidate mediators, respectively. 95% confidence intervals with 5,000 bootstrapped resamples estimated the magnitude and significance of indirect effects. Multicollinearity diagnostics using Values Inflation Factors (VIF) were run prior to mediation tests to assess any inflation in the variance of estimated regression coefficients, with a threshold value of 3.3 [66].

## Results

### Descriptive analyses

Children’s mean trait resilience score at 10.5 years old was 59.97 (*SD* = 17.14; Range = 5–100). Skewness was -0.32, suggesting an approximately symmetric distribution with slight negative skew, with slightly more children rating themselves on the higher end of resilience. A kurtosis score of -0.09 denoted a distribution that was slightly more peaked and with heavier tails than a normal distribution, but still within a range of moderate deviation from normality.

Girls (*M* = 59.2, *SD* = 17.9) and boys (*M* = 60.6, *SD* = 16.4) had comparable resilience scores at age 10.5 years, *t*(428) = 0.85, *p* = .40, 95% CI [-4.66, 1.85]. Meanwhile, ethnicity was significantly associated with children’s resilience, *F*(2, 420) = 5.65, *p* = .004, with a small-to-medium effect size, *η*² = 0.03. Specifically, children of Indian ethnicity endorsed higher resilience (*M* = 67.3, *SD* = 17.5), relative to Chinese (*M* = 59.6, *SD* = 17.5, *p* = .01) and Malay (*M* = 57.8, *SD* = 15.7, *p* = .003) children. Neither marital status nor maternal and paternal ages at baseline were significantly associated with children’s trait resilience at age 10.5 years, *p*s > 0.10. Given our central interest in early SES characteristics, ensuing analyses adjusted for ethnicity to minimize confounding effects of demographics in the role of socioeconomic characteristics on children’s trait resilience.

### Zero-order associations between socioeconomic characteristics and children’s resilience

Maternal education at baseline was significantly associated with group mean differences in children’s trait resilience at age 10.5 years, *F*(2, 415) = 10.25, *p* < .001. The eta-squared value was *η*² = 0.05, corresponding to a small-to-medium effect size. Post-hoc pairwise tests with Bonferroni correction demonstrated that children whose mothers had completed up to a secondary level of education (*M* = 55.0, *SD* = 16.3) endorsed less trait resilience, compared to those whose mothers were had post-secondary diplomas (*M* = 60.0, *SD* = 17.4, *p* = .043), or who were college-educated (*M* = 64.3, *SD* = 16.8, *p* < .001); polynomial contrasts revealed that this relationship was linear in nature, *F*(1) = 10.07, *p* < .001. Likewise, paternal education was significantly associated with group differences, *F*(2, 344) = 4.70, *p* = .01, with a small-to-medium effect size, *η*² = 0.03. Children whose fathers were college-educated (*M* = 63.2, *SD* = 17.3) reported higher resilience scores at age 10.5 years than counterparts who had attained up to a secondary level of education (*M* = 56.2, *SD* = 16.9, *p* = .007); the effect of paternal education demonstrated a linear pattern, *F*(1) = 4.66, *p* = .002. In parallel, we observed a significant effect of household income, *F*(3, 391) = 5.21, *p* = .002, with a small-to-medium effect size, *η*² = 0.04. Children with monthly household incomes more than S$6,000 exhibited higher resilience (*M* = 64.0, *SD* = 16.7) compared to those in the less than S$2000 category (*M* = 53.9, *SD* = 16.5, *p* = .001). Similarly, the relationship of household income with children’s trait resilience was revealed to be linear, *F*(1) = 5.06, *p* < .001. By contrast, housing ownership was not significantly predictive of children’s resilience, *F*(2, 413) = 1.35, *p* = .26. Supplementary Table 2 summarizes resilience scores by demographic and socioeconomic characteristics [Additional File 4].

### Mediation analyses

Little’s (1998) test was significant, χ2(297) = 385.00, *p* < .001, suggesting that missing patterns were not completely at random. In light of this, the ensuing mediational analyses employed Full Information Maximum Likelihood (FIML) estimations to leverage all available information to produce unbiased estimates without listwise deletion.

In the maternal analysis, maternal education and household income (baseline) were entered as focal predictors, and maternal parenting dimensions (maternal warmth, rejection, and autonomy support at 8.5 years) were parallel mediators. Analogously, the paternal analysis was conducted with paternal education and household income (baseline) as focal predictors, and paternal warmth, rejection, and autonomy support (measured at 8.5 years) as concurrent mediators. Analyses adjusted for children’s ethnicity (1 = *Indian* or 0 = *non-Indian*). VIF values of SES predictors and mediators were acceptable at 1.13–1.65 for maternal analysis and 1.15–1.46 for paternal analysis [66]. 

**Fig. 1:**
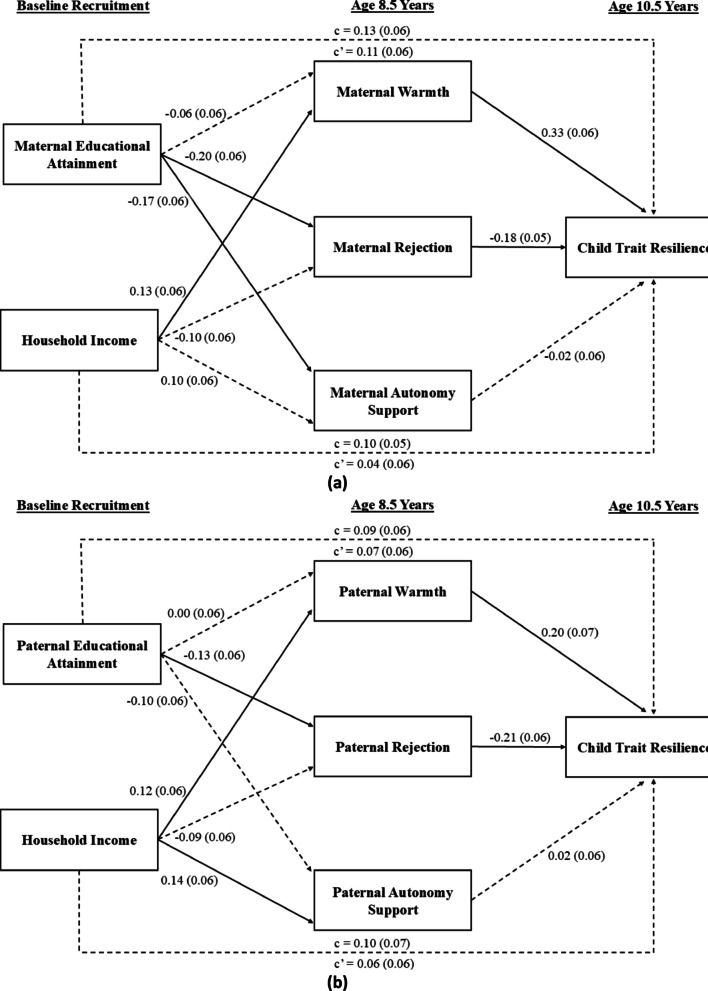
(**a**) Standardized Beta Coefficients and Standard Errors of Maternal Education, Maternal Warmth, Rejection and Autonomy Support, and Children’s Resilience (*N* = 1339). (**b**) Standardized Beta Coefficients and Standard Errors of Paternal Education, Paternal Warmth, Rejection and Autonomy Support, and Children’s Resilience (*N* = 746). *Note*. Numbers outside of brackets represent standardized beta coefficients, and numbers inside brackets represent standard errors of these estimates. Solid lines represented significant paths, whereas dotted lines represented non-significant paths. Estimates were rounded to 2 decimal places. Models adjusted for child ethnicity (1 = Indian, 0 = Non-Indian). FIML estimations were used to maximize all available information and obtain unbiased parameter estimates. *c* represents the total effect of SES predictors on trait resilience, capturing both the direct influence and any indirect influence mediated through parenting. *c*’ denotes the direct effect of SES predictors on trait resilience after statistically accounting for parenting, isolating that which is left unexplained by mediating pathways

In the maternal analysis (Fig. 1a), higher maternal education at baseline predicted less maternal rejection at age 8.5 years,= *β* = -0.20, *SE* = 0.06, 95%CI[-0.31, -0.08], and less autonomy support, *β* = -0.17, *SE* = 0.06, 95%CI[-0.29, -0.05], but was not significantly associated with maternal warmth, *β* = -0.06, *SE* = 0.06, 95%CI[-0.18, 0.07]. Lower levels of maternal rejection at age 8.5 years in turn predicted greater child resilience at age 10.5 years, *β* = -0.18, *SE* = 0.05, 95% CI[-0.28, -0.08]. Meanwhile, the path from maternal autonomy support to children’s resilience was not statistically significant, *β* = -0.02, *SE* = 0.06, 95%CI[-0.12, 0.09]. Overall, the indirect effect of maternal education on children’s resilience was significant through maternal rejection, *β* = 0.04, *SE* = 0.01, 95%CI[0.01, 0.06], but not maternal warmth, *β* = -0.018, *SE* = 0.02, 95%CI[-0.06, 0.02], nor maternal autonomy support, *β* = 0.00, *SE* = 0.01, 95%CI[-0.02, 0.02]. The initial significant total effect of maternal education (*β* = 0.13, *SE* = 0.06, 95%CI[0.01, 0.25]) turned non-significant upon inclusion of the three mediators, Direct Effect: *β* = 0.11, *SE* = 0.06, 95%CI[-0.01, 0.22], suggesting full mediation.

Meanwhile, higher household income at baseline predicted more maternal warmth at age 8.5 years, *β* = 0.13, *SE* = 0.06, 95%CI[0.01, 0.24], but was neither associated with maternal rejection, *β* = -0.10, *SE* = 0.06, 95%CI[-0.21, 0.02], nor autonomy support, *β* = 0.10, *SE* = 0.06, 95%CI[-0.02, 0.22]. Higher levels of maternal warmth at age 8.5 years were, in turn, linked to higher child resilience at age 10.5 years, *β* = 0.33, *SE* = 0.06, 95%CI[0.22, 0.44]. Overall, the indirect effect of household income was significant through maternal warmth, *β* = 0.04, *SE* = 0.02, 95%CI[0.00, 0.08], but not maternal rejection, *β* = 0.02, *SE *= 0.01, 95%CI[-0.01, 0.04], or autonomy support, *β* = 0.00, *SE* = 0.01, 95%CI[-0.01, 0.01]. Upon entering the three mediators, the initial total effect of household income (*β* = 0.10, *SE* = 0.05, 95%CI[-0.02, 0.21]) attenuated in strength, Direct Effect: *β* = 0.04, *SE* = 0.06, 95%CI [-0.07, 0.15]. Maternal analysis accounted for 22.6% of inter-individual variance in resilience.

In the paternal analysis (Fig. 1b), higher paternal education at baseline likewise predicted less paternal rejection at age 8.5 years, *β* = -0.13, *SE* = 0.06, 95%CI[-0.25, -0.01]. By contrast, paternal education was not predictive of paternal warmth, *β* = 0.00, *SE* = 0.06, 95%CI [-0.12, 0.12], nor paternal autonomy support, *β* = -0.10, *SE* = 0.06, 95%CI[-0.23, 0.02]. Similarly to mothers, lower levels of paternal rejection at age 8.5 years then significantly predicted greater child resilience at age 10.5 years, *β* = -0.21, *SE* = 0.06, 95%CI[-0.32, -0.09]. There was a significant indirect effect of paternal education on children’s resilience though paternal rejection, *β* = 0.03, *SE* = 0.01, 95%CI [0.00, 0.06], but not paternal warmth, *β* = 0.00, *SE* = 0.01, 95%CI[-0.02, 0.02], or paternal autonomy support, *β* = 0.00, *SE* = 0.01, 95%CI[-0.01, 0.01]. The initial total effect of paternal education (*β* = 0.09, *SE* = 0.06, 95%CI[-0.04, 0.22]) attenuated in strength upon accounting for the three mediators, Direct Effect: *β* = 0.07, *SE* = 0.06, 95%CI[-0.06, 0.19]. Meanwhile, higher household income predicted greater paternal warmth, *β* = 0.12, *SE* = 0.06, 95%CI[0.00, 0.24]. and greater paternal autonomy support, *β* = 0.14, *SE* = 0.06, 95%CI[0.02, 0.27], but not paternal rejection, *β* = -0.09, *SE* = 0.06, 95%CI[-0.21, 0.03]. While greater paternal warmth was in turn predictive of children’s greater trait resilience at age 10.5 years, *β* = 0.20, *SE* = 0.07, 95%CI[0.07, 0.33], paternal autonomy support was not, *β* = 0.02, *SE* = 0.06, 95%CI [-0.11, 0.14]. Indirect effects of household income via paternal warmth was marginally significant, *β* = 0.02, *SE* = 0.01, 95%CI[-0.01, 0.05], and non-significant via paternal autonomy support, *β* = 0.00, *SE* = 0.01, 95%CI[-0.02, 0.02]. Overall, paternal analysis accounted for 14.7% of inter-individual variance in resilience.

To evaluate robustness of mediation effects, we conducted supplementary sensitivity analyses adjusting for the caregiver-reported Social Emotional Assets and Resilience Scale (SEARS) [67] at age 7 years. The SEARS captures children’s social-emotional functioning in terms of self-regulation, social competence, and empathy. These traits have been empirically linked to trait resilience [68] as well as parents’ caregiving practices [69]. Mediation results were found to be robust to covariate adjustment using the SEARS, with the direction and significance of key pathways unchanged, suggesting that the observed mediational effects were not driven by children’s prior social-emotional adjustment (see Supplementary Analysis 2 and Supplementary Table 3 for full details [Additional File 5]).

## Discussion

Using a longitudinal mediation approach, we investigated indirect pathways from early socioeconomic conditions to trait resilience in late childhood through maternal and paternal parenting dimensions in middle childhood. We found that both parents’ education at baseline was prospectively linked with child resilience at age 10.5 years through parents’ rejection behaviors at age 8.5 years; meanwhile, household income at baseline predicted trait resilience at age 10.5 years through maternal warmth at age 8.5 years. Altogether, SES disadvantage early in life set the stage for greater parental rejection behaviors and less maternal warmth behaviors during middle childhood, consequently attenuating children’s resilience in late childhood.

The average trait resilience score of Singaporean children at 10.5 years old was 59.97 (*SD* = 17.14). Given comparatively high safety within Singapore [70], this mean score was surprising and purportedly low in comparison to CD-RISC-25 scores in community samples of 13-year-olds in Canada (*M* = 63.22, SD = 13.05) [71] and 14-year-olds in China (*M* = 70.63, *SD* = 13.20) [62]. Future research is merited to disambiguate what drives the heterogeneity in resilience scores across countries; likely, a combination of maturational processes (psychological maturity of younger versus older children), cultural niche factors (e.g., high academic stress), and sampling discrepancies, may be at play. Correspondingly, the use of multilevel modelling presents one way to distinguish within-person effects (age) from group-level effects (culture).

### Theoretical and translational implications

The present longitudinal study advances theoretical literature in three principal ways. Firstly, the present study lends empirical support to the generalizability of the FSM to Singapore’s cultural milieu [43]. Specifically, we pinpointed key indices of socioeconomic risk preceding children’s trait resilience in Singapore’s ecology. With respect to household income, the only significant group difference observed was that of households with < S$2,000 monthly income, compared to counterparts with monthly incomes >S$6,000; with children from the latter having higher trait resilience than the former. This pattern suggests that policy interventions that prioritize helping families achieve economic advancement beyond the S$2,000 threshold (e.g., income supplement) may potentially yield pronounced gains in children’s trait resilience. With respect to both maternal and paternal education, children whose parents had completed up to a secondary level of schooling exhibited diminished trait resilience compared to counterparts who had higher educational levels (e.g., post-secondary diplomas and college education). Likewise, this pattern suggests that structural initiatives to support parental attainment beyond secondary education (e.g., enhanced schooling subsidies for parents with only secondary education) could potentially mitigate SES-associated trait resilience disparities at this level. In contrast, housing type was not a significant SES predictor. Given extensive housing grants, majority of Singaporean families live in government-subsidized public flats. Consequently, housing type might lack the granularity necessary to distinguish SES within Singapore’s landscape.

Secondly, we identified specific family-level processes arising sequentially from socioeconomic risk that underpin the path from SES to children’s trait resilience: parents from disadvantaged SES backgrounds in Singapore may unwittingly adopt unsupportive parenting practices corresponding with less warmth and greater rejection, curtailing children’s trait resilience development. Corroborating with a cross-sectional study where parental warmth predicted Turkish preschool children’s trait resilience [72], we extended this line of inquiry by uncovering comparable relationships in middle-to-late childhood in Singapore using a longitudinal design, offering insight that parental warmth may be promotive of trait resilience development beyond early preschool years. Interestingly, parental autonomy support appeared to be inconsequential to children’s trait resilience in our study. Findings here diverge from past research that parental autonomy support is beneficial in bolstering Euro-American children’s social-emotional adjustment [24]. Several counterfactuals warrant discussion to understand this discrepancy and null finding. A first plausible reason pertains to measurement error. The Cronbach’s alphas of 0.75 to 0.76 for mothers’ and fathers’ autonomy support indexed only a modest level of internal consistency based on rule-of-thumb scientific thresholds [73]. This suggests the items may not be fully homogenous in capturing autonomy support within our Singaporean sample, potentially attenuating the strength of observed associations. Another reason could be cross-cultural distinctions in the underlying developmental function of parental autonomy support [74]. As explicated by Bao and Lam [75], Asian children's experiences of autonomy are shaped by the social values and relational structures of collectivist cultures (e.g., interdependent self-construals). More precisely, Asian children may derive a sense of autonomy from the social-emotional connectedness they share with close caregivers who make decisions for them, rather than through having personal freedom [75]. Given that the PBI frames autonomy support in line with Anglo-centric concepts of self-reliance, the items may not have captured Asian children’s perceived parental autonomy support. A pivotal step for future research is to utilize emic methodologies that prioritize bottom-up perspectives in the design of culturally-tailored autonomy support measurement tools.

Thirdly, while mothers have well-documented influences on children’s social-emotional health [76], our present research suggests that fathers’ parenting evinced parallel associations as mothers’ parenting, albeit to a weaker extent. This spotlights the fairly overlooked role of fathers in the socialization process, a comparatively underexplored area in family process literature [77]. 

Taken altogether, our findings accentuate the benefits of having structured parenting supports to actively strengthen the early caregiving practices of parental figures facing socioeconomic risk. For one, Attachment-Based Family Therapy may be an empirically supported treatment that can foster reparative parent-child relationships via the mitigation of parental rejection [78]. Meanwhile, a burgeoning body of translational work [79] has suggested that parenting-focused mindfulness-based interventions may be one promising avenue to augment maternal warmth [80, 81]. As a next step, feasibility research would be warranted to discern the acceptability and validity of these strategies for Singaporean households facing SES-associated stress.

### Strengths and limitations

As noted by Putnick and colleagues [82], past studies on parental rejection have been correlational or cross-sectional, stymying inferences on causality and directionality. A notable strength of the study was the longitudinal design over three timepoints, which furnishes the benefit of observing temporal sequences. Another strength lies in the ability to disentangle the influence of SES, a multifaceted construct, via the use of individual indices, illuminating how specific SES conditions operate through somewhat distinctive pathways to shape children’s resilience.

Nevertheless, several limitations merit discussion. First, a caveat lies in shared method bias, given that parenting and child resilience measures were reported by the same informant (i.e., child). Future research could leverage multi-informant assessments with observed or parent-reported measures. We balance this perspective with a caution that parent-reported parenting measures are frequently inflated as parents tend to present themselves as more supportive than children perceive them to be [83]. It is further worth noting that children’s perspectives are attested to be more ecologically valid and reflective of the lived experiences of the child [76]. 

Second, as previously described, structural constraints in enrolling biological fathers only in the later phases of GUSTO engendered a systematic under-representation of paternal participants from the “<$2,000” income category. This pattern of SES-associated non-response may reflect challenges inherent with socioeconomic stress such as time demands and less flexible working arrangements [84] and suggests that a degree of caution is necessitated when interpreting our results pertaining to paternal influences. Notably, selection bias linked to fathers’ SES is a well-documented challenge within family literature [84]. Future research could seek to leverage targeted recruitment strategies, such as building partnerships with social service agencies that may be well-positioned to facilitate research engagement among fathers facing economic risk.

Another key methodological challenge in parenting science, and in our study, lies in disaggregating maternal and paternal influences, whose parenting practices are highly correlated (see Supplementary Table 4 [Additional File 6]). To advance understanding about unique roles of mothers and fathers in child development, future research could investigate strategies to isolate these influences, such as with statistical approaches informed by the Actor–Partner Interdependence Model [85]. 

Moreover, only 14.7% to 22.6% of inter-individual variance in children’s trait resilience was accounted for. The small-to-medium effect sizes (η² = 0.03–0.05) further suggest additional explanatory factors remain unidentified and would be an important avenue for future work. At the individual level, children who experience parental rejection may struggle with emotional regulation and self-esteem, potentially compromising the capacity to adaptively confront emotional stressors [86]. At the interpersonal level, environmental influences beyond home—such as teacher and peer support—be complementary interpersonal influences alongside parenting in shaping children’s trait resilience [87], in keeping with social-ecological models of child development [88]. Additionally, our findings only partially substantiate the FSM, as only some pathways were evaluated. Specifically, parental mental health and interparental conflict—key tenets of the FSM—were not directly tested [43]. Future research could thus seek to replicate and extend the current model to more fully elucidate environmental antecedents to children’s trait resilience.

Finally, while our study shed light on the developmental function of parental warmth in Singapore, we utilized standardized parenting tools that were originally formulated from within Anglo-White populations: although conferring the benefit of cross-study comparability, these were limited in capturing culturally moderated manifestations of parental warmth. An influential study illustrates this patently: Chinese immigrant mothers in the United States conveyed warmth not just through affection, but through attending to children’s daily routines and the provision of guidance on learning [89]. More broadly, the parenting discipline is undergoing a paradigmatic shift in favor of emic methods—those that foreground and unravel how caregiving is conceived, enacted, and ascribed meaning within the parameters of local value systems [30]. In line with this shift, future work on the familial antecedents of trait resilience could adopt culturally adapted questionnaires alongside qualitative techniques (e.g., open-ended interviews) to uncover culturally specific caregiving practices shaped by socioeconomic constraints in Singapore households. Such an approach would accentuate both the cultural sensitivity and empirical precision of future scholarship in this area.

## Conclusions

This longitudinal study highlighted that children raised under early conditions of socioeconomic adversity—marked by limited household income and parental education—were more likely to experience greater parental rejection and diminished maternal warmth during middle childhood—key relational experiences that then circumscribed the formation of trait resilience in late childhood. In doing so, we extended an increasing body of empirical research to support the pertinence and validity of the FSM within an understudied Southeast Asian family ecology. Findings further emphasized a need to address both structural SES inequalities alongside enhancing supportive caregiving practices to foster children’s trait resilience.

## Supplementary Information

Below is the link to the electronic supplementary material.

Supplementary Material 1.

Supplementary Material 2.

Supplementary Material 3.

Supplementary Material 4.

Supplementary Material 5.

Supplementary Material 6.

## Data Availability

The GUSTO data are not publicly available due to multi-site partnership agreements and conditions for Internal Review Board approval. GUSTO data are however routinely made available through submission and approval of data access requests routed to the cohort executive committee. Information may be obtained from the corresponding author upon reasonable request.

## Abbreviations

ANOVA: Analyses of Variance
CD-RISC-25: Connor-Davidson Resilience Scale-25
FIML: Full Information Maximum Likelihood
FSM: Family Stress Model
GUSTO: Growing Up in Singapore Towards Healthy Outcomes
MCAR: Missing Completely at Random
PBI: Parental Bonding Instrument
SEARS: Social Emotional Assets and Resilience Scale
SES: Socioeconomic Status
VIF: Values Inflation Factors
