# Supplementary material for: Early socioeconomic conditions to children’s trait resilience: longitudinal mediation effects of mothers’ and fathers’ parenting
Source: Child Adolesc Psychiatry Ment Health. 2025 Nov 10;19:123. doi: 10.1186/s13034-025-00979-1 (PMC12604427; doi:10.1186/s13034-025-00979-1)
Supplement: Supplementary file 1 — Supplementary Material 1. [file 13034_2025_979_MOESM1_ESM.docx]

**Supplementary Table 1**
Sample Characteristics (*N* = 430)

| **Variable** | ***N*** | **Mean (SD)** | **Percentage (%)** |
| --- | --- | --- | --- |
| **Child’s Biological Sex** |  |  |  |
| Female | 206 |  | 47.9 |
| Male | 224 |  | 52.1 |
| **Child Ethnicity** |  |  |  |
| Chinese | 225 |  | 57.3 |
| Malay | 118 |  | 30.0 |
| Indian | 50 |  | 12.7 |
| **Maternal Age at Child’s Birth** |  | 30.5 (5.13) |  |
| 18 to 24 (Young Mothers) | 57 |  | 13.4 |
| 25 to 34 (Average Childrearing Ages) | 261 |  | 61.6 |
| 35 and Above (Advanced Parental Age) | 106 |  | 25.0 |
| **Paternal Age at Child’s Birth** |  | 33.9 (6.03) |  |
| 18 to 24 (Young Fathers) | 10 |  | 3.0 |
| 25 to 34 (Average Childrearing Ages) | 177 |  | 52.2 |
| 35 and Above (Advanced Parental Age) | 152 |  | 44.8 |
| **Parents’ Marital Status** |  |  |  |
| Single | 14 |  | 3.38 |
| Married | 400 |  | 96.62 |
| **Maternal Education** |  |  |  |
| Secondary or Below | 125 |  | 29.8 |
| Diploma or Certificates | 152 |  | 36.3 |
| College and Above | 142 |  | 33.9 |
| **Paternal Education** |  |  |  |
| Secondary or Below | 94 |  | 27.0 |
| Diploma/Certificates | 122 |  | 35.1 |
| College and Above | 132 |  | 37.9 |
| **Household Monthly Income (Singapore Dollar)** |  |  |  |
| < S$2000 | 63 |  | 15.9 |
| S$2000 to S$3999 | 115 |  | 29.0 |
| S$4000 to S$5999 | 99 |  | 25.0 |
| >S$6000 | 119 |  | 30.1 |
| **Housing Type** |  |  |  |
| 1-, 2-,or 3-Room Public Flat | 103 |  | 24.7 |
| 4- or 5-Room or Executive Public Flat | 282 |  | 67.6 |
| Private Property | 32 |  | 7.7 |

***Note****.* Maternal and paternal ages were collected as continuous variables, but were re-coded into ordinal categories for ease of interpretability and in anticipation of non-linear effects. We present both the mean and standard deviation, as well as percentage breakdown by category for maternal and paternal ages.
