# Supplementary material for: Early socioeconomic conditions to children’s trait resilience: longitudinal mediation effects of mothers’ and fathers’ parenting
Source: Child Adolesc Psychiatry Ment Health. 2025 Nov 10;19:123. doi: 10.1186/s13034-025-00979-1 (PMC12604427; doi:10.1186/s13034-025-00979-1)

**Supplementary Analysis 1**

**Differences Between Families With and Without Paternal Participants**

To ascertain the degree of sampling bias associated with the non-participant of biological fathers in some families, we conducted supplementary analyses comparing whether families *with* paternal participants (*N* = 348, coded as 1) systematically differed from counterparts *without* paternal participants (*N* = 82, coded as 0).

Welch’s *t*-test revealed no systematic differences in children’s trait resilience scores in families with versus without paternal indicators, *t*(123.74) = 0.81, *p* = .42. Likewise, children’s ratings of fathers’ warmth, rejection, and autonomy support were not significantly different for fathers who participated versus those who did not, Warmth: *t*(81.98)= 0.79, *p* = .43; Rejection: *t*(84.17) = 0.54, *p* = .59; Autonomy Support: *t*(86.62) = 0.58, *p* = .57.

A chi-square test of independence further denoted that the distribution of children’s ethnicity and gender were not systematically different in these groups, Ethnicity: χ²(2, 423) = 1.66*, p* = .44; Gender: χ²(1, 430) = 0.003*, p* = .96.

Moreover, SES as proxied by maternal education levels was not systematically different in the two groups, χ²(2, 419) = 1.03, *p* = 0.60. A significant difference was revealed in terms of household income levels, χ²(3, 396) = 13.681, *p* = .003. An examination of standardized residuals denoted that families with missing paternal indicators were over-represented in the lowest-income category (Standardized Residual = 3.34) and under-represented in the “$4000 to $5999” category (Standardized Residual = -2.15), suggesting that that the selection bias was driven primarily by disproportionality in these income categories (see Supplementary Figure 1).

**Supplementary Figure 1**
Proportion of Families With and Without Paternal Participants by Household Income Group


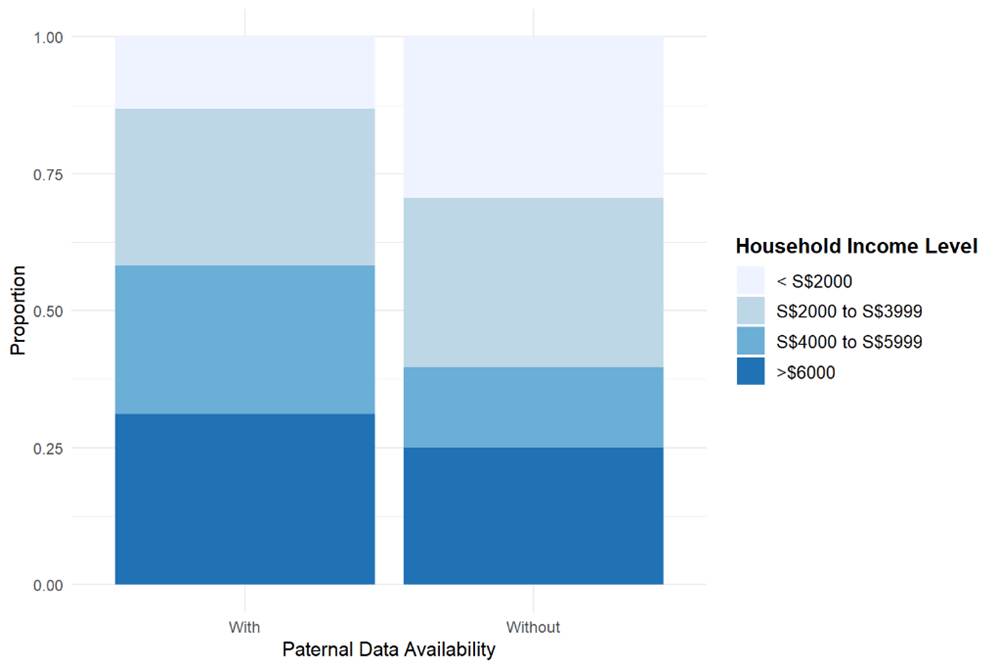

Supplement: Supplementary file 2 — Supplementary Material 2. [file 13034_2025_979_MOESM2_ESM.docx]
