# Supplementary material for: Early socioeconomic conditions to children’s trait resilience: longitudinal mediation effects of mothers’ and fathers’ parenting
Source: Child Adolesc Psychiatry Ment Health. 2025 Nov 10;19:123. doi: 10.1186/s13034-025-00979-1 (PMC12604427; doi:10.1186/s13034-025-00979-1)

**Supplementary Figure 2**

1. Scatterplot of Maternal Age Plotted Against Children’s Trait Resilience (*N* = 424)


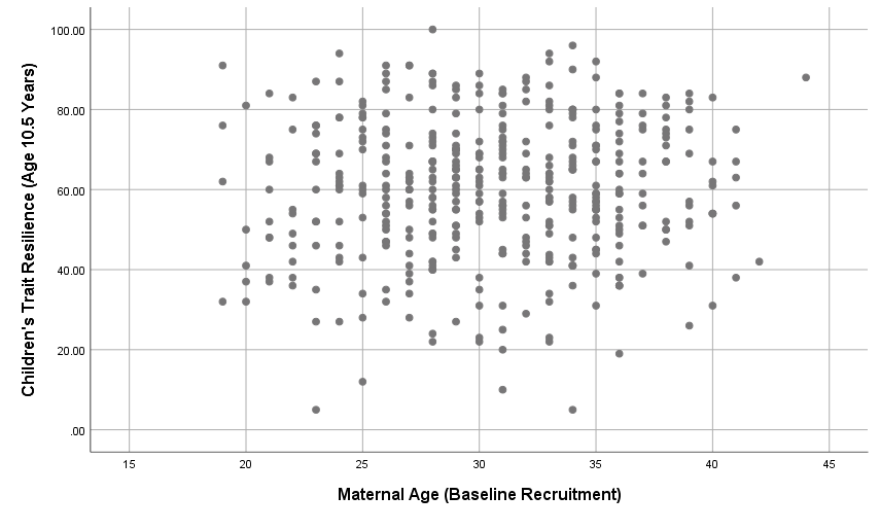


1. Scatterplot of Paternal Age Plotted Against Children’s Trait Resilience (*N* = 339)


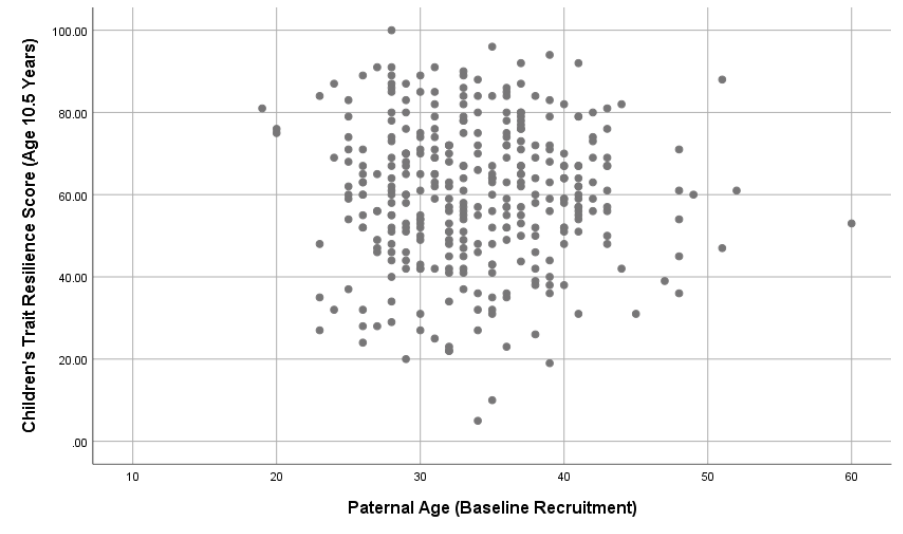

Supplement: Supplementary file 3 — Supplementary Material 3. [file 13034_2025_979_MOESM3_ESM.docx]
