# Supplementary material for: Early socioeconomic conditions to children’s trait resilience: longitudinal mediation effects of mothers’ and fathers’ parenting
Source: Child Adolesc Psychiatry Ment Health. 2025 Nov 10;19:123. doi: 10.1186/s13034-025-00979-1 (PMC12604427; doi:10.1186/s13034-025-00979-1)
Supplement: Supplementary file 4 — Supplementary Material 4. [file 13034_2025_979_MOESM4_ESM.docx]

**Supplementary Table 2**
Children’s CD-RISC-25 Scores by Socioeconomic and Demographic Variables (*N* = 430)

| **Demographic Characteristic** | ***N*** | **Mean Resilience Score (SD)** | **Test Statistics (df)** | ***p*** |
| --- | --- | --- | --- | --- |
| **Child’s Biological Sex** |  |  | *t*(428) = 0.85 | .40 |
| Female | 206 | 59.2 (17.9) |  |  |
| Male | 224 | 60.6 (16.4) |  |  |
| **Child Ethnicity** |  |  | *F*(2, 420) = 5.65, *η*² = 0.03 | .004 |
| Chinese | 225 | 59.6 (17.5) |  |  |
| Malay | 118 | 57.8 (15.7) |  |  |
| Indian | 50 | 67.3 (17.5) |  |  |
| **Maternal Age at Child’s Birth** |  |  | *F*(2, 421) = 0.85, *η*² < 0.01 | .43 |
| 18 to 24 (Young Mothers) | 57 | 57.3 (18.7) |  |  |
| 25 to 34 (Average Childrearing Ages) | 261 | 60.4 (17.6) |  |  |
| 35 and Above (Advanced Parental Age) | 106 | 60.6 (15.4) |  |  |
| **Paternal Age at Child’s Birth** |  |  | *F*(2, 336) = 0.06, *η*² < 0.01 | .94 |
| 18 to 24 (Young Fathers) | 10 | 61.4 (23.4) |  |  |
| 25 to 34 (Average Childrearing Ages) | 177 | 59.9 (17.7) |  |  |
| 35 and Above (Advanced Parental Age) | 152 | 60.5 (16.1) |  |  |
| **Parents’ Marital Status** |  |  |  |  |
| Single | 14 | 57.6 (18.5) | *t*(412) = 0.52 | .61 |
| Married | 400 | 60.1 (17.2) |  |  |
| **Socioeconomic Characteristic** | ***N*** | **Mean Resilience Score (SD)** | **Test Statistics (df)** | ***p*** |
| **Maternal Education** |  |  | *F*(2, 415) = 10.25, *η*² = 0.05 | < .001 |
| Secondary or Below | 125 | 55.0 (16.3) |  |  |
| Diploma or Certificates | 152 | 60.0 (17.4) |  |  |
| College and Above | 142 | 64.3 (16.8) |  |  |
| **Paternal Education** |  |  | *F*(2, 344) = 4.70, *η*² = 0.03 | .01 |
| Secondary or Below | 94 | 56.2 (16.9) |  |  |
| Diploma/Certificates | 122 | 60.4 (16.9) |  |  |
| College and Above | 132 | 63.2 (17.3) |  |  |
| **Household Monthly Income (Singapore Dollar)** |  |  | *F*(3, 391) = 5.21, *η*² = 0.04 | .002 |
| < S$2000 | 63 | 53.9 (16.5) |  |  |
| S$2000 to S$3999 | 115 | 59.8 (17.6) |  |  |
| S$4000 to S$5999 | 99 | 58.9 (16.7) |  |  |
| >S$6000 | 119 | 64.0 (16.7) |  |  |
| **Housing Type** |  |  | *F*(2, 413) = 1.35, *η*² < 0.01 | .26 |
| 1-, 2-,or 3-Room Public Flat | 103 | 57.6 (18.6) |  |  |
| 4- or 5-Room or Executive Public Flat | 282 | 60.6 (16.7) |  |  |
| Private Property | 32 | 61.5 (18.1) |  |  |

***Note****.* * *p* < .05, ** *p* < .01, *** *p* < .001. Analyses of socioeconomic characteristics adjusted for children’s ethnicity (coded as 1 = Indian, 0 = Non-Indian).
