# Supplementary material for: Early socioeconomic conditions to children’s trait resilience: longitudinal mediation effects of mothers’ and fathers’ parenting
Source: Child Adolesc Psychiatry Ment Health. 2025 Nov 10;19:123. doi: 10.1186/s13034-025-00979-1 (PMC12604427; doi:10.1186/s13034-025-00979-1)
Supplement: Supplementary file 5 — Supplementary Material 5. [file 13034_2025_979_MOESM5_ESM.docx]

**Supplementary Analysis 2**

**Sensitivity Analyses for the Mediation Effects of Mothers’ and Fathers’ Parenting on Children’s Trait Resilience**

To evaluate the robustness of mediation effects, we conducted supplementary analyses adjusting for the caregiver-reported Social Emotional Assets and Resilience Scale (SEARS)^1^ as an additional covariate. The SEARS was developed specifically as a parent report of children’s social-emotional functioning along the dimensions of self-regulation (22 items: “*Stays calm when there is a problem or argument*”), social competence (10 items: e.g., “I*s comfortable talking to many different people*”, and empathy (7 items: e.g., “*Tries to help others when they need help*”). The dimensions have been empirically associated with the development of trait resilience^2-4^ as well as in the prediction of parents’ caregiving practices,^5,6^ thereby serving a meaningful control to isolate the influence of parenting above and beyond children’s baseline social-emotional functioning.

Items were rated on a 4-point Likert scale from 0 = “*Never*” to 3 = “*Always*”. No item-level missingness was observed. Item-level scores were summed to form a composite social-emotional functioning score, where higher scores reflected better functioning. It was administered to children’s biological mothers (91.90%), biological fathers (6.08%), or a biological grandparent (2.03%) during the in-person study visit at age 7 years. Internal consistency in the present sample was excellent (Cronbach’s α = 0.96).

The pattern of mediation results stayed consistent and were robust to covariate adjustment using the SEARS, with the direction and significance of key pathways unchanged. The results suggest that the observed mediation effects were not driven by children’s baseline social-emotional adjustment.

**Supplementary Table 3.**
Standardized Beta Coefficients, Standard Errors, and 95% Confidence Intervals of Mediation Paths with Additional Covariate Adjustment

| **Pathway** | ***β*** | **SE** | **95%CI** |
| --- | --- | --- | --- |
| **Indirect Effects via Maternal Parenting (*N* = 369)** | | | |
| Maternal Education → Maternal Warmth → Trait Resilience | -0.02 | 0.02 | [-0.06, 0.02] |
| Maternal Education → Maternal Rejection → Trait Resilience | 0.04 | 0.02 | [0.01, 0.07]* |
| Maternal Education → Maternal Autonomy Support → Trait Resilience | 0.00 | 0.01 | [-0.02, 0.03] |
| Household Income → Maternal Warmth → Trait Resilience | 0.04 | 0.02 | [0.01, 0.09]* |
| Household Income → Maternal Rejection → Trait Resilience | 0.01 | 0.01 | [-0.01, 0.03] |
| Household Income → Maternal Autonomy Support → Trait Resilience | 0.00 | 0.01 | [-0.02, 0.01] |
| **Indirect Effects via Paternal Parenting (*N* = 309)** | | | |
| Paternal Education → Paternal Warmth → Trait Resilience | 0.00 | 0.01 | [-0.03, 0.02] |
| Paternal Education → Paternal Rejection → Trait Resilience | 0.03 | 0.02 | [0.00, 0.06]* |
| Paternal Education → Paternal Autonomy Support → Trait Resilience | 0.00 | 0.01 | [-0.02, 0.02] |
| Household Income → Paternal Warmth → Trait Resilience | 0.02 | 0.03 | [-0.06, 0.05] |
| Household Income → Paternal Rejection → Trait Resilience | 0.02 | 0.01 | [-0.01, 0.04] |
| Household Income → Paternal Autonomy Support → Trait Resilience | 0.00 | 0.01 | [-0.02, 0.02] |

***Note.*** Asterisks denote significant indirect effects based on 95% confidence intervals. Estimates were rounded to 2 decimal places. Models adjusted for child ethnicity (1 = *Indian*, 0 = *Non-Indian*) as well as parent-reports of children’s social-emotional functioning, measured using the Social Emotional Resilience and Assets Scale at age 7 years. Full Information Maximum Likelihood (FIML) estimation was used.

**References**

1. Merrell KW, Felver-Gant JC, Tom KM. Development and validation of a parent report measure for assessing social-emotional competencies of children and adolescents. J Child Fam Stud. 2011;20(4):529-40. <https://doi.org/10.1007/s10826-010-9425-0>.
2. Artuch-Garde R, González-Torres MdC, de la Fuente J, Vera MM, Fernández-Cabezas M, López-García M. Relationship between resilience and self-regulation: a study of Spanish youth at risk of social exclusion. Front Psychol. 2017;8:612. <https://doi.org/10.3389/fpsyg.2017.00612>.
3. Nakhostin-Khayyat M, Borjali M, Zeinali M, Fardi D, Montazeri A. The relationship between self-regulation, cognitive flexibility, and resilience among students: a structural equation modeling. BMC Psychol. 2024;12(1). <https://doi.org/10.1186/s40359-024-01843-1>.
4. Wang J, Yang Q, Yu X, Hu L. Effects of adolescent empathy on emotional resilience: the mediating role of depression and self-efficacy and the moderating effect of social activities. Behav Sci. 2024;14(3):228. <https://doi.org/10.3390/bs14030228>.
5. Isdahl-Troye A, Villar P, Álvarez-Voces M, Romero E. Unraveling the dynamics of emotional regulation and parental warmth across early childhood: prediction of later behavioral problems. Sci Rep. 2025;15(1):23294. <https://doi.org/10.1038/s41598-025-06846-5>.
6. Strayer J, Roberts W. Children’s anger, emotional expressiveness, and empathy: relations with parents’ empathy, emotional expressiveness, and parenting practices. Soc Dev. 2004;13(2):229-54. <https://doi.org/10.1111/j.1467-9507.2004.000265.x>.
