# Supplementary material for: Early socioeconomic conditions to children’s trait resilience: longitudinal mediation effects of mothers’ and fathers’ parenting
Source: Child Adolesc Psychiatry Ment Health. 2025 Nov 10;19:123. doi: 10.1186/s13034-025-00979-1 (PMC12604427; doi:10.1186/s13034-025-00979-1)
Supplement: Supplementary file 6 — Supplementary Material 6. [file 13034_2025_979_MOESM6_ESM.docx]

**Supplementary Table 4.**

Correlation Matrix of Study Variables (*N* = 430)

|  | *N* | 1 | 2 | 3 | 4 | 5 | 6 | 7 | 8 | 9 | 10 | 11 | 12 | 13 | 14 |
| --- | --- | --- | --- | --- | --- | --- | --- | --- | --- | --- | --- | --- | --- | --- | --- |
| 1. Child Resilience | 430 | — |  |  |  |  |  |  |  |  |  |  |  |  |  |
| 2. Maternal Education | 419 | 0.21 *** | — |  |  |  |  |  |  |  |  |  |  |  |  |
| 3. Paternal Education | 348 | 0.16  ** | 0.60  *** | — |  |  |  |  |  |  |  |  |  |  |  |
| 4. Household Monthly Income | 396 | 0.17 *** | 0.60 *** | 0.57 *** | — |  |  |  |  |  |  |  |  |  |  |
| 5. Housing Type | 417 | 0.07 | 0.21 *** | 0.23 *** | 0.43 *** | — |  |  |  |  |  |  |  |  |  |
| 6. Child Sex | 430 | 0.04 | 0.02 | 0.04 | 0.07 | -0.02 | — |  |  |  |  |  |  |  |  |
| 7. Child Ethnicity | 393 | 0.16  ** | 0.11* | 0.17  ** | -0.05 | -0.12  * | 0.01 | — |  |  |  |  |  |  |  |
| 8. Maternal Age at Child’s Birth | 424 | 0.05 | 0.14  ** | 0.12* | 0.15  ** | 0.19  *** | - 0.01 | -0.11* | — |  |  |  |  |  |  |
| 9. Paternal Age at Child’s Birth | 339 | 0.01 | 0.04 | 0.04 | 0.13* | 0.14* | - 0.01 | -0.04 | 0.57 *** | — |  |  |  |  |  |
| 10. Maternal Warmth | 340 | 0.34 *** | 0.03 | 0.00 | 0.09 | 0.05 | -0.07 | 0.08 | -0.07 | -0.09 | — |  |  |  |  |
| 11. Maternal Rejection | 340 | -0.27 *** | -0.27 *** | -0.25 *** | -0.23 *** | -0.17  ** | 0.06 | 0.00 | -0.07 | -0.06 | -0.1 | — |  |  |  |
| 12. Maternal Autonomy Support | 340 | 0.09 | -0.1 | -0.09 | -0.04 | -0.03 | -0.08 | 0.00 | -0.07 | -0.1 | 0.51 *** | 0.08 | — |  |  |
| 13. Paternal Warmth | 338 | 0.33 *** | 0.09 | 0.07 | 0.13* | 0.11* | -0.03 | 0.13* | -0.06 | -0.02 | 0.57 *** | -0.23 *** | 0.26 *** | — |  |
| 14. Paternal Rejection | 338 | -0.26 *** | -0.19 *** | -0.19  ** | -0.19 *** | -0.15  ** | 0.06 | -0.01 | -0.1 | -0.13* | -0.14* | 0.73 *** | 0 | -0.31 *** | — |
| 15. Paternal Autonomy Support | 338 | 0.18  ** | 0.00 | -0.03 | 0.03 | -0.02 | -0.06 | 0.09 | -0.05 | 0.03 | 0.31 *** | -0.02 | 0.69 *** | 0.49 *** | -0.09 |

***Note****.* Asterisks denote statistically significant *p* -values; * *p* < .05, ** *p* < .01, *** *p* < .001. Pairwise deletion was used in cases of missing data. Child sex was coded as 1 = male, 0 = female. Child ethnicity was coded as 1 = Indian, 0 = non-Indian.
